# Editorial: Soil microbe-arthropod interactions under global change

**DOI:** 10.3389/fmicb.2023.1280103

**Published:** 2023-09-19

**Authors:** Rentao Liu, Bing Yang

**Affiliations:** ^1^School of Ecology and Environment, Ningxia University, Yinchuan, China; ^2^Sichuan Academy of Giant Panda, Chengdu, China

**Keywords:** soil microbiology, soil arthropod, soil microbe-arthropod interactions, global change, ecosystem functionality

In recent decades, the field of ecology has witnessed a growing recognition of the intricate relationships that exist within ecosystems. One such dynamic interaction exists between soil microbial communities and soil arthropods, two vital components of terrestrial ecosystems that play fundamental roles in nutrient cycling, decomposition, and overall ecosystem functioning (Bardgett et al., 1998). Increasing evidences suggest that soil biota and their interactions are sensitive to global changes, and the effects of interacting global change factors on soil biotic activity have been a widespread ecological focus (Crowther et al., 2015). To better understand the cascade effects of various types of environmental stresses on ecosystem multifunctionality, deciphering the keystone species that play key roles in the stabilization of ecosystem under stressful environments, we need to develop reliable knowledge for soil biotic responses to our changing world.

To date, the responses of soil microbial communities (Castro et al., 2010; Zhou et al., 2020, 2023; Abs et al., 2023; Baldrian et al., 2023; Cruz-Paredes et al., 2023; Hu et al., 2023; Jansson and Wu, 2023; Jia et al., 2023; Meena et al., 2023) and/or soil microarthropods (Liu et al., 2020; Meehan et al., 2020; Barreto et al., 2023; Thakur et al., 2023) have been intensively studied. As suggested, the interactions between soil microorganisms and soil invertebrates include direct predator-prey relationships, but also indirect effects, such as competition for resources and habitat formation (Scheu et al., 2005). The interactions between soil microbial communities and soil microarthropods are expected to be affected by global changes (Crowther et al., 2015). In general, ecosystems with strong and balanced interactions between soil microbes and soil arthropods are better equipped to withstand environmental stresses and maintain their functionality. These suggest that a comprehensive understanding of how these two vital components of ecosystems influence each other and contribute to overall ecosystem resilience under various environmental stresses is of utmost importance.

In this Research Topic, we explored the responses of soil microbial communities, soil microarthropods and their interactions to a wide range of global changes. Wang et al. used field experiment to examine changes in soil microbial diversity, composition, and functional genes in response to experimentally warming and increasing precipitation across two growing seasons in a temperate semiarid grassland of the Horqin region. They found that warming exerted negative effects on soil microbial diversity, composition, and putative functional genes associated with carbon and nitrogen cycles, whereas increasing precipitation weakened the negative effects of simulated warming on soil microbial diversity. They demonstrated that bacterial and fungal diversities respond consistently to the global change scenario in semiarid sandy grassland, but the underlying mechanisms were distinct. The authors suggested that the co-occurrence of warming and increasing precipitation could alleviate the negative effects of global change on biodiversity loss and ecosystem degradation under a predicted climate change scenario in a semiarid grassland.

Pen-Mouratov and Dayan quantified the abundance, trophic structure, sex ratio and genus diversity of soil free-living nematodes, and total abundances of bacteria and fungi during the wet and dry seasons in the nesting and roosting habitats of the following piscivorous and omnivorous colonial birds, including black kite (*Milvus migrans*), great cormorant (*Phalacrocorax carbo*), black-crowned night heron (*Nycticorax nycticorax*) and little egret (*Egretta garzetta*), in Israel's Mediterranean region. They observed that the different species of colonial birds could result in different abundance, diversity, and community structure of the soil free-living nematodes at the generic, trophic and sexual levels during the wet and dry seasons. It worth noting that seasonal fluctuation that could attenuate the effect of bird activity on the abundance, and the structure and diversity of the soil communities.

Zhang et al. investigated the differences in the diversity indices and community composition of fungal community in rhizosphere soils of monoculture and mixture plantation stands of arbuscular mycorrhizal (AM) tree species (*Fraxinus mandschurica*) and ectomycorrhizal (EM) tree species (*Larix gmelinii, Picea koraiensis*) in Northeastern China. They found that the changes in soil physicochemical properties and litter quality accounted for the observed differences in mycorrhizal colonization rate, composition, and diversity of the rhizosphere mycorrhizal community, especially the EMF community. They observed closely associations between EMF community and soil moisture, pH, nitrate nitrogen content, dissolved organic nitrogen content, soil organic matter content, soil organic carbon/total nitrogen and litter carbon/total nitrogen. These results provided a better understanding of the effects of plant-soil feedbacks on ecological functioning, and casted a new light on the importance of selecting tree species and establishing plant community in ecological restoration of degraded ecosystem.

Tian et al. aimed to identify a novel bacterial biocontrol against *Meloidogyne incognita*, an obligate parasitic nematode with a wide variety of hosts that causes huge economic losses every year via assaying the nematocidal activity of *Bacillus velezensis* strain Bv-25 obtained from cucumber rhizosphere. They found that the strain Bv-25 exhibited direct nematocidal against *M. incognita in vitro*, suppressed gene expression of *M. incognita* J2s as a fumigant, and remarkably reduced nematode infection in laboratory trials, with the evidence promoting gene expression in the salicylic acid and jasmonic acid signaling pathways, consequently improving cucumber resistance against *M. incognita*. By using pot combined with field trials, the authors indicated that the strain Bv-25 significantly decreased the disease index of *M. incognita* and increased cucumber yield. They concluded that *B. velezensis* strain Bv-25 has good potential to control root-knot nematodes. It was suggested that strain Bv-25 had multiple anti-nematode properties that justify its application in biocontrol against *M. incognita*.

Overall, this Research Topic provides a diverse glimpse into how soil biotic communities and associated ecosystem functioning would be changed under global change in the future. Some specific mechanisms that govern the relationships between soil microbial communities and arthropods has been uncovered. While these studies have shed light on individual aspects of these interactions, a comprehensive synthesis of knowledge, highlighting both current understandings and critical knowledge gaps, is currently lacking. Further research is needed to understand the mechanistic underpinnings of these interactions, especially in the context of rapid global change. Integrating molecular techniques, advanced imaging, and long-term monitoring could enhance our understanding of these complex relationships. These can develop more informed strategies for managing and conserving ecosystems in the face of global change, promoting their long-term sustainability and the services they provide to humans and the environment.

## Author contributions

RL: Conceptualization, Funding acquisition, Writing—original draft, Writing—review and editing. BY: Writing—review and editing.

## References

[B1] Abs E. Chase A. B. Allison S. D. (2023). How do soil microbes shape ecosystem biogeochemistry in the context of global change? Environ. Microbiol. 25, 780–785. 10.1111/1462-2920.16331 36579433

[B2] Baldrian P. López-Mondéjar R. Kohout P. (2023). Forest microbiome and global change. Nat. Rev. Microbiol. 21, 487–501. 10.1038/s41579-023-00876-4 36941408

[B3] Bardgett R. D. Keiller S. Cook R. Gilburn A.S. (1998). Dynamic interactions between soil animals and microorganisms in upland grassland soils amended with sheep dung: a microcosm experiment. Soil Biol. Biochem. 30, 531–539. 10.1016/S0038-0717(97)00146-6

[B4] Barreto C. Conceição P. H. S. de Lima E. C. A. Stievano L. C. Zeppelini D. Kolka R. K.. (2023). Large-scale experimental warming reduces soil faunal biodiversity through peatland drying. Front. Environ. Sci. 11, 508. 10.3389/fenvs.2023.1153683

[B5] Castro H. F. Classen A. T. Austin E. E. Norby R. J. Schadt C. W. (2010). Soil microbial community responses to multiple experimental climate change drivers. Appl. Environ. Microbiol. 76, 999–1007. 10.1128/AEM.02874-09 20023089 PMC2820983

[B6] Crowther T. W. Thomas S. M. Maynard D. S. Baldrian P. Covey K. Frey S. D.. (2015). Biotic interactions mediate soil microbial feedbacks to climate change. PNAS 112, 7033–7038. 10.1073/pnas.1502956112 26038557 PMC4460469

[B7] Cruz-Paredes C. Tájmel D. Rousk J. (2023). Variation in temperature dependences across Europe reveals the climate sensitivity of soil microbial decomposers. Appl. Environ. Microbiol. 89, e02090–22. 10.1128/aem.02090-22 37162342 PMC10231190

[B8] Hu Y. L. Ganjurjav H. Hu G. Z. Wang X. X. Wan Z. Q. Gao Q. Z. (2023). Seasonal patterns of soil microbial community response to warming and increased precipitation in a semiarid steppe. Appl. Soil Ecol. 182, 104712. 10.1016/j.apsoil.2022.104712

[B9] Jansson J. K. Wu R. N. (2023). Soil viral diversity, ecology and climate change. Nat. Rev. Microbiol. 21, 296–311. 10.1038/s41579-022-00811-z 36352025

[B10] Jia M. Q. Gao Z. W. Huang J. Li J. Liu Z. Y. Zhang G. G.. (2023). Soil bacterial community is more sensitive than fungal community to nitrogen supplementation and climate warming in Inner Mongolian desert steppe. J. Soils Sed. 23, 405–421. 10.1007/s11368-022-03283-z

[B11] Liu R. T. Navon Y. Steinberger Y. Sternberg M. (2020). Effects of rainfall manipulations vs. a natural aridity gradient on plant litter arthropods in desert and Mediterranean ecosystems. Appl. Soil Ecol. 156, 103716. 10.1016/j.apsoil.2020.103716

[B12] Meehan M. L. Barreto C. Turnbull M. S. Bradley R. L. Bellenger J. P. Darnajoux R.. (2020). Response of soil fauna to simulated global change factors depends on ambient climate conditions. Pedobiologia 83, 150672. 10.1016/j.pedobi.2020.150672

[B13] Meena M. Yadav G. Sonigra P. Nagda A. Mehta T. Swapnil P.. (2023). Multifarious responses of forest soil microbial community toward climate change. Microb. Ecol. 86, 49–74. 10.1007/s00248-022-02051-3 35657425

[B14] Scheu S. Ruess L. Bonkowski M. (2005). “Interactions between microorganisms and soil micro- and mesofauna,” in Soil Biology, Microorganisms in Soil: Roles in Genesis and Functions V3, eds Buscot F. Varma A. (Springer, Berlin), 253-274.

[B15] Thakur M. P. Sigurðsson B. D. Sigurðsson P. Holmstrup M. (2023). Warming shifts the biomass distribution of soil microarthropod communities. Soil Biol. Biochem. 177, 108894. 10.1016/j.soilbio.2022.108894

[B16] Zhou S. Y. D. Lie Z. Y. Liu X. J. Zhu Y. G. Penuelas J. Neilson R.. (2023). Distinct patterns of soil bacterial and fungal community assemblages in subtropical forest ecosystems under warming. Glob. Chang. Biol. 29, 1501–1513. 10.1111/gcb.16541 36448266

[B17] Zhou Z. H. Wang C. K. Luo Y. Q. (2020). Meta-analysis of the impacts of global change factors on soil microbial diversity and functionality. Nat. Commun. 11:3072. 10.1038/s41467-020-16881-7 32555185 PMC7300008

